# Rabies Virus Infection Induces the Formation of Stress Granules Closely Connected to the Viral Factories

**DOI:** 10.1371/journal.ppat.1005942

**Published:** 2016-10-17

**Authors:** Jovan Nikolic, Ahmet Civas, Zoé Lama, Cécile Lagaudrière-Gesbert, Danielle Blondel

**Affiliations:** Institute for Integrative Biology of the Cell (I2BC), CEA, CNRS, Univ Paris-Sud, Université Paris-Saclay, Gif-sur-Yvette, France; Harvard Medical School, UNITED STATES

## Abstract

Stress granules (SGs) are membrane-less dynamic structures consisting of mRNA and protein aggregates that form rapidly in response to a wide range of environmental cellular stresses and viral infections. They act as storage sites for translationally silenced mRNAs under stress conditions. During viral infection, SG formation results in the modulation of innate antiviral immune responses, and several viruses have the ability to either promote or prevent SG assembly. Here, we show that rabies virus (RABV) induces SG formation in infected cells, as revealed by the detection of SG-marker proteins Ras GTPase-activating protein-binding protein 1 (G3BP1), T-cell intracellular antigen 1 (TIA-1) and poly(A)-binding protein (PABP) in the RNA granules formed during viral infection. As shown by live cell imaging, RABV-induced SGs are highly dynamic structures that increase in number, grow in size by fusion events, and undergo assembly/disassembly cycles. Some SGs localize in close proximity to cytoplasmic viral factories, known as Negri bodies (NBs). Three dimensional reconstructions reveal that both structures remain distinct even when they are in close contact. In addition, viral mRNAs synthesized in NBs accumulate in the SGs during viral infection, revealing material exchange between both compartments. Although RABV-induced SG formation is not affected in MEFs lacking TIA-1, TIA-1 depletion promotes viral translation which results in an increase of viral replication indicating that TIA-1 has an antiviral effect. Inhibition of PKR expression significantly prevents RABV-SG formation and favors viral replication by increasing viral translation. This is correlated with a drastic inhibition of IFN-B gene expression indicating that SGs likely mediate an antiviral response which is however not sufficient to fully counteract RABV infection.

## Author Summary

Exposure of cells to environmental stresses, such as heat shock and viral infection, induces a cellular response leading to the formation of Stress Granules (SGs) composed of stalled translation initiation complexes (RNA-binding proteins and mRNA). The subsequent inhibition of host translation participates to cell survival. Viruses modulate or interfere with SG formation to control viral replication and antiviral responses, but differences exist in the dynamics and outcome of the stress responses induced by various viruses. Our study shows that Rabies virus (RABV) induces the formation of SGs in infected cells. We combined different methods of advanced imaging techniques (live-cell imaging, 3D analysis, FISH experiments) to characterize for the first time these structures. SGs are highly dynamic structures that increase in size by fusion events, exhibit transient assembly or persist throughout infection. They localize close to viral factories, cytoplasmic structures characteristic of RABV infection involved in viral replication and transcription. Viral messenger RNAs, but not viral genomic RNA, are transported from the factories to SGs, indicating the communication between both compartments. In addition, we provide some evidence that RABV-induced cellular stress is dependent on double-stranded RNA-activated protein kinase (PKR). Our data indicate that PKR also participates in innate immune responses through the induction of Interferon-B gene. Taken together, our results give an insight on new and important aspects of RABV infection and host antiviral stress responses.

## Introduction

Viral infections initiate a number of cellular stress responses that modulate gene expression by affecting the regulation of cellular mRNA translation, localization and degradation, while promoting viral transcription, replication and translation [1]. One of the stress responses is the assembly of messenger ribonucleoprotein (mRNP) complexes into dynamic cytoplasmic structures known as stress granules (SGs) and processing bodies (P bodies) [2–5].

Viruses also modify cellular gene expression by initiating the transcriptional activation of type I interferon (IFN) genes and interferon-stimulated genes (ISGs) that mediate antiviral responses [6]. During viral infection, viral RNAs are recognized by different pattern recognition receptors (PRR), such as RIG-I and MDA5. This recognition triggers a series of events leading to the activation of protein kinase R (PKR) and the subsequent initiation of the SGs assembly [7–10]. Activated PKR mediates translation inhibition upon replication of many RNA viruses [7] by phosphorylating the eukaryotic initiation factor-2 regulatory subunit (eIF2 α). Inhibition of eIF2 α activity interferes with the formation of eIF2-GTP-Met-tRNAi Met ternary complex required for the delivery of initiator Met-tRNAi to the 40S ribosomal subunit thereby stalling the translation initiation of most mRNAs [11]. Subsequent reduction of protein synthesis promotes cellular survival by limiting the consumption of energy and nutrients, and reallocating resources to the repair of cellular damages. During PKR-induced SGs formation, specific RNA-binding proteins with self-aggregating properties, such as ras GTPase-activating protein-binding protein 1 (G3BP1), T-cell intracellular antigen 1 (TIA-1), and TIA-1-related protein (TIAR), recruit translationally inactive messenger ribonucleoproteins (mRNPs) and these complexes nucleate the formation of SGs [12]. During this process, the poly(A)-binding protein (PABP) is also sequestered into SGs [13, 14]. After the stress removal, the mRNAs are released for translation on ribosomes or degradation in P-bodies [13, 15, 16].

It has become clear that a growing number of viruses, particularly RNA viruses, modulate RNA granule formation and function to maximize replication efficiency [1]. Different consequences of viral infection on these structures and their components have been described: they include induction of SGs (which is most often transient), complete inhibition of SGs formation, and alternate SGs assembly and disassembly during the course of infection. Several mechanisms are used by viruses to interfere with SGs assembly. Influenza A virus blocks SGs formation throughout infection by expressing the NS1 protein, which suppresses IFN activation and serves as a potent PKR antagonist through its dsRNA binding activity [17]. Some positive RNA viruses encode proteases which cleave key SGs components leading to the disassembly of SGs [18]. Others divert SG factors into viral replication complexes to favor viral replication likely at the expense of SGs formation [19, 20]. Among negative RNA viruses, vesicular stomatitis virus (VSV) produces SG–like structures containing TIA1 and TIAR that appear similar to transcription and replication inclusions as they contain viral replicative proteins and viral RNAs [21, 22]. Thus, some viruses actively induce SG formation and utilize the stress response for their own benefit while some others inhibit SG formation, which suggests an antiviral function for these structures.

Rabies virus (RABV), the prototype of the *Lyssavirus* genus that, as VSV, belongs to the *Rhabdoviridae* family, causes a fatal disease that is associated with intense viral replication in the central nervous system. The viral genome consisting of single-stranded negative-sense RNA (~12kb), encodes five proteins, the nucleoprotein (N), the phosphoprotein (P), the matrix protein (M), the glycoprotein (G), and the polymerase (L). The virus enters the host cell through the endosomal transport pathway via a low-pH-induced membrane fusion process catalyzed by G. Viral transcription and replication take place within Negri bodies (NBs), which are cytoplasmic inclusions bodies formed during viral infection as viral factories [23]. During transcription, a positive–strand leader RNA and five capped and polyadenylated mRNAs are synthesized. The replication process yields nucleocapsids containing full-length antigenome-sense RNA which in turn serves as template for the synthesis of viral genomic RNA. After additional rounds of transcription and/or replication, neo-synthesized RNPs are transported to the cell membrane where they are assembled with the M and G proteins into virions, which are then released from the cell through the budding process.

In this report, we show that during infection, RABV promotes the formation of cytoplasmic SGs that contain TIA-1, G3BP1 and PABP. Using live cell imaging of infected cells, we also show that these structures are highly dynamic during the viral cycle. RABV-induced SGs are distinct, but close to Negri bodies as revealed by the physical proximity of both structures and by the transport of viral mRNA from NBs into SGs. The role of PKR and TIA-1 in SG formation, viral replication and IFN-B gene expression is also investigated. Our observations indicate that RABV induces PKR-dependent cell stress and innate immune responses.

## Results

### RABV infection induces SGs formation

**Fig 1:**
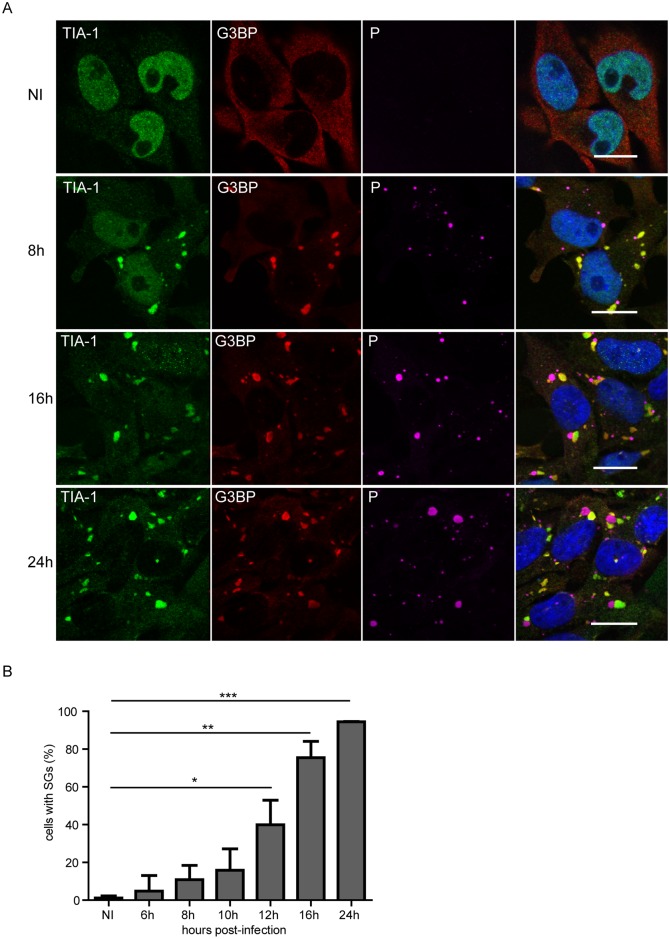
RABV infection induces the formation of SG-like structures. (A) U373-MG cells grown on glass coverslips were either non-infected (NI) or infected with RABV (CVS strain) at a MOI of 3. At different times post-infection (8 h, 16 h, 24 h), cells were analyzed by confocal microscopy after co-staining with the goat anti-TIA-1, mouse anti-G3BP1 and rabbit anti-P antibodies followed by incubation with Alexa-488 donkey anti-goat IgG, Alexa-568 donkey anti-mouse IgG and Alexa-647 donkey anti-rabbit IgG. DAPI (blue) was used to stain the nuclei (merge). Colocalization of TIA1 and G3BP1 is apparent as yellow coloration in the merged panel. The scale bars correspond to 15 μm. (B) U373-MG were infected with RABV as above for the indicated times. The percentage of cells containing SG was quantified as described in Materials and Methods (left panel). Each time point represents the average of three independent experiments. p values (* p <0.05; ** p < 0.01; *** p<0.001) were determined using an impaired Student *t* test. Error bars show standard deviations.

**Fig 2:**
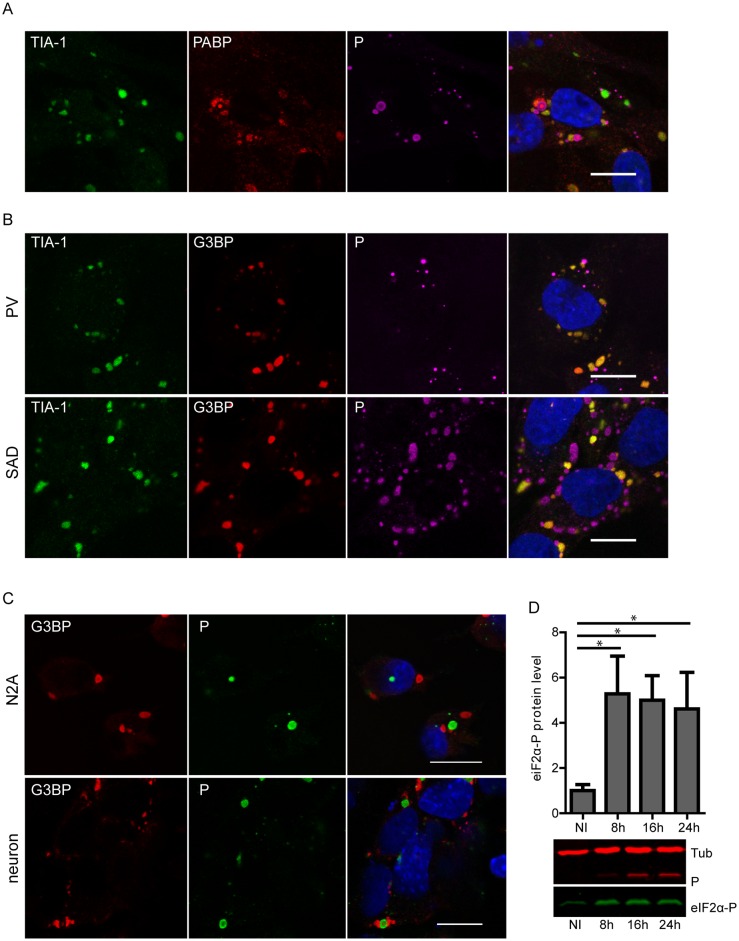
RABV induced-SGs contain PABP and SGs formation is independent of virus strain and cell-type. (A) U373-MG cells were infected with RABV (CVS strain) at a MOI of 3. At 24 h p.i, cells were analyzed by confocal microscopy after co-staining with the goat anti-TIA-1, mouse anti-PABP and rabbit anti-P antibodies followed by incubation with Alexa-488 donkey anti-goat IgG, Alexa-568 donkey anti-mouse IgG and Alexa-647 donkey anti-rabbit IgG. DAPI (blue) was used to stain the nuclei (merge). Colocalization of TIA-1 and PABP is apparent as yellow coloration in the merged panel. The scale bars correspond to 15 μm. (B) U373-MG cells were either infected with RABV (PV strain) or (SAD B19) at a MOI of 3. At 24 h p.i, cells were analyzed by confocal microscopy after co-staining with the goat anti-TIA-1, mouse anti-G3BP1 and the rabbit anti-P antibodies followed by incubation with Alexa-488 donkey anti-goat IgG, Alexa-568 donkey anti-mouse IgG and Alexa-647 donkey anti-rabbit IgG. DAPI (blue) was used to stain the nuclei (merge). Colocalization of TIA-1 and G3BP1 is apparent as yellow coloration in the merged panel. The scale bars correspond to 15 μm. (C) Cortical neurons prepared as described in material and methods, and N2A cells were infected by RABV (CVS strain). At 24 h p.i cells were analyzed by confocal microscopy after co-staining with the mouse anti-G3BP1 and rabbit anti-P antibodies followed by incubation with Alexa-568 goat anti-mouse and Alexa-488 goat anti-rabbit IgG. DAPI (blue) was used to stain the nuclei (merge). The scale bars correspond to 15 μm. (D) U373-MG cells were either non-infected (NI) or infected with RABV (CVS strain) at a MOI of 3. At different time p.i, cell extracts were analyzed by western blot using anti-phospho-eIF2α, anti-P and anti-α-tubulin antibodies. Western blots from three independent experiments were quantified using immunoblot scanning and normalized with respect to the amount of α-tubulin. The amount of phosphorylated eIF2α was measured in comparison to an arbitrary level value of 1 applied to protein levels obtained in non-infected cells. Significant difference between infected and non-infected cells was determined using a Student *t* test and denoted by asterisks (* p < 0.05). Error bars indicate the standard deviation.

Given the role of SGs in virus-induced stress responses, we investigated whether RABV infection induces the formation of SGs in infected cells. For this analysis, we used human and mouse neuroblastoma cell lines and primary neurons isolated from mice. Human glioblastoma U373-MG cells were infected with RABV (CVS strain) at a MOI of 3 for various times (up to 24 h). Cells were then immuno-stained for G3BP1 and TIA-1 that are well-established SG-associated proteins used as SG markers [24]. Immunofluorescence microscopy revealed that, following infection, TIA-1 translocated from the nucleus to the cytoplasm and formed aggregates co-localizing with G3BP1 (Fig 1A, first and second column), indicating that SGs are formed in RABV-infected cells. In contrast, SGs were not observed in uninfected cells over the time-course of the experiment. Co-immunostaining of infected cells with antibodies specific for RABV P protein that mainly localizes within the NBs revealed that NBs and SGs were distinct structures formed during viral infection (Fig 1A). The kinetics of SG formation during viral infection showed that SGs were detectable at 6–8 h p.i. The number of infected cells containing SGs increased throughout the 24 h period of infection (Fig 1B). Another SG-marker, poly(A)-binding protein (PABP), colocalized with TIA-1-containing foci confirming the formation of SGs during RABV infection, although PABP was sometimes detected in NBs (Fig 2A).

SG induction was not specific to RABV-CVS strain, since two other RABV strains, PV and SAD B19, also induced SG formation in infected cells (Fig 2B). SGs were also observed in mouse neuroblastoma cells (N2A cells) and primary neurons infected by RABV (Fig 2C), indicating that SG formation is a general process triggered upon RABV infection.

To show that the stress response pathway of translation inhibition is activated following RABV infection, we then analyzed eIF2α phosphorylation status that is closely linked to SG formation and usually initiated by PKR activation in virus-infected cells [1, 9, 25] RABV enhanced eIF2α phosphorylation in infected cells. The level of phosphorylated eIF2α is already maximal at 8 h p.i (around 5 fold) and remained at the same level thereafter (Fig 2D). This is consistent with the kinetics of SG formation (Fig 1A).

### Formation and dynamics of SGs induced in response to RABV infection

**Fig 3:**
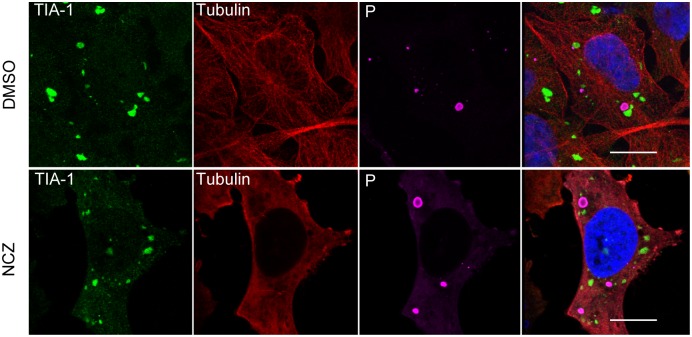
Effect of nocodazole on RABV-induced SGs. U373-MG cells were infected and treated with nocodazole (NCZ) (2 μM) or mock treated (DMSO) for 1 h before virus inoculation and during infection. At 20 h p.i as indicated, cells were co-stained with goat anti-TIA-1, mouse anti-α tubulin and rabbit anti-P antibodies, followed by incubation with a mixture of Alexa-488 donkey anti-goat IgG, Alexa-568 donkey anti-mouse IgG and Alexa-647 donkey anti-rabbit IgG. The scale bars correspond to 15 μm.

It has been reported that disruption of the microtubule network by drugs such as nocodazole or vinblastine affects SGs formation [26, 27]. We therefore examined the role of microtubule network, in the formation of SGs following RABV infection. As shown in Fig 3, depolymerization of microtubules with nocodazole, confirmed by anti-tubulin labelling, did not impair the formation of SGs in RABV-infected cells. These results indicated that the formation of SGs in RABV-infected cells did not require an intact microtubule network.

**Fig 4:**
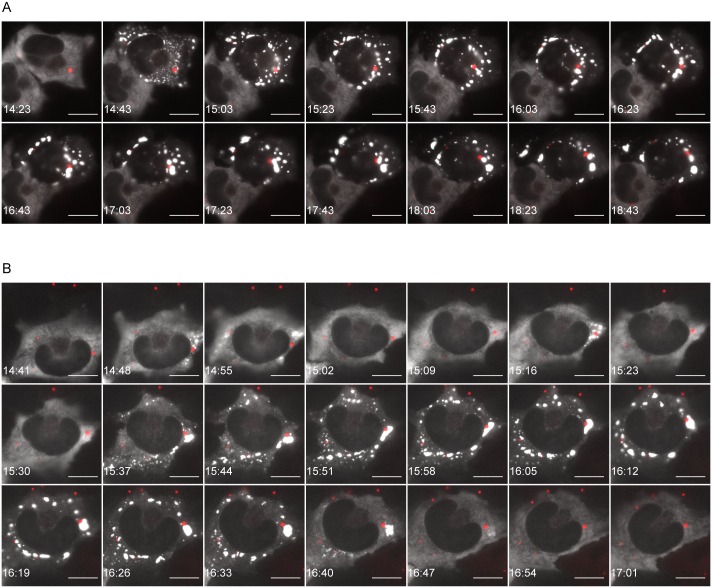
Live cell imaging of RABV infected cells. U373-MG cells were transiently transfected with pG3BP-eGFP (to visualize SGs) and infected for 14 h with the recombinant virus rCVSN2C-P-mCherry. Live-cell time-lapse experiments were performed on ten cells. Seven cells (out of ten) exhibit a persistent pattern (one cell shown in A); three cells (out of ten) exhibit a transient pattern (one cell shown in B). G3BP-GFP signals (white) and P-mCherry signals (red) are merged. The time post-infection is displayed in the lower left corner of each panel and the scale bars correspond to 15 μm. Movies from which images have been extracted correspond to S1 and S2 Movies.

To monitor the process of SGs assembly in living cells, U373-MG cells were transiently transfected with an expression plasmid for G3BP1-eGFP and infected for 14 h with the recombinant virus rCVSN2C-P-mcherry (obtained as described in Material and Methods). In non-infected cells, G3BP1-GFP staining was diffuse and cytoplasmic, while, as expected, most of the infected cells contained SGs (S1 Fig). In cells infected with the recombinant virus (revealed by their inner red data punctuate structures), SGs formation was observed with G3BP1 relocalization in small cytoplasmic granules that increased in size over time (Fig 4). Analysis of infected cells by video-microcopy and time-lapse fluorescence (images were acquired every 1 min during 7 h) showed that SGs are highly dynamic structures which fuse together when in close contact (Fig 4, and S1 and S2 Movies in supplemental data). Some cells (seven out of ten cells) contained large SGs which were localized close to NBs and persisted for more than four hours (Fig 4A). In other cells (three out of ten cells), small size SGs, transiently formed at the vicinity of NBs, exhibited alternate cycles of assembly-disassembly, and finally gave rise to larger SGs (Fig 4B). In both cases, RABV-induced SGs growing over time by fusion events upon contact exhibited characteristic liquid droplet behavior of non-membrane bound intracellular RNA granules [28–30].

**Fig 5:**
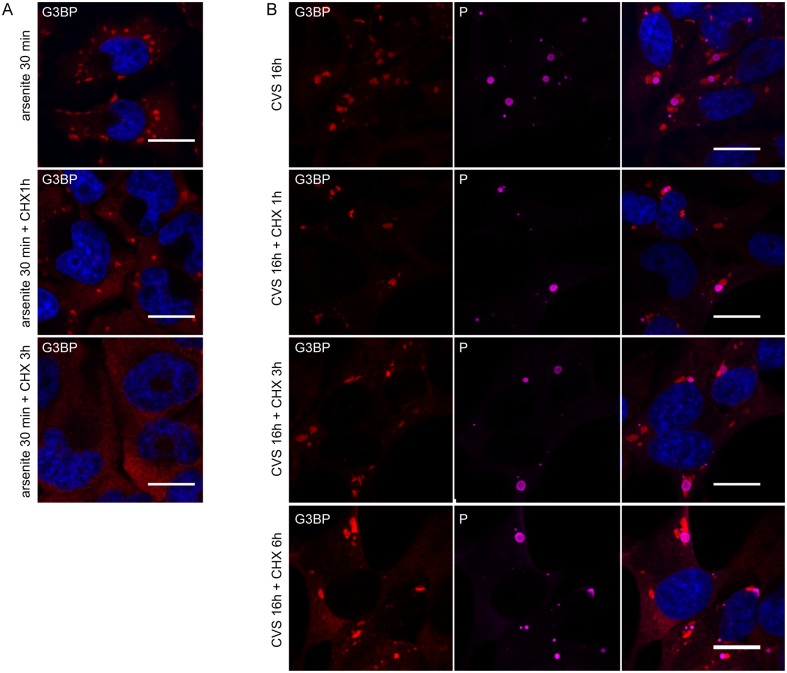
SGs induced by arsenite are disrupted by the protein synthesis inhibitor CHX whereas RABV-induced SGs are not. (A) U373-MG cells were treated with arsenite (0.5mM) for 30 min and subsequently were either left untreated or treated with CHX for another 1 h or 3 h (as indicated) before processing for IF. Cells were then stained for G3BP1. DAPI (blue) was used to stain the nuclei (merge). The scale bars correspond to 15 μm. (B) U373-MG cells were infected with CVS for 16 h before IF (top panel) or were treated with CHX at 16 h p.i for 1 h or 3 h or 6 h as indicated and were then processed for IF to detect P and G3BP proteins. Staining with secondary antibodies and DAPI was carried as in Fig 1. The scale bars correspond to 15 μm.

We next explored whether viral protein synthesis is required for the maintenance of the SGs in infected cells. Cycloheximide (CHX), a translational inhibitor of protein synthesis, was used in infected cells to examine the formation of SGs. CHX has been shown to be an inhibitor of SGs formation, since it traps mRNAs in polysomes by blocking translational elongation and thus preventing the formation and/or maintenance of SGs [14]. As expected, the SGs induced by sodium arsenite were disrupted when cells were incubated with CHX for 1 h or 3 h (Fig 5A). In contrast, in RABV infected cells, no major change in the number and the size of SGs was observed at 16 h p.i after incubation of infected cells with CHX for an additional 1 h or 3 h or 6 h (Fig 5B). This result indicated that the maintenance of RABV-induced SGs does not require viral and/or cellular translation. These data altogether revealed that RABV-induced SGs have specific features and are not canonical SGs.

### SGs and NBs are distinct structures in close proximity

**Fig 6:**
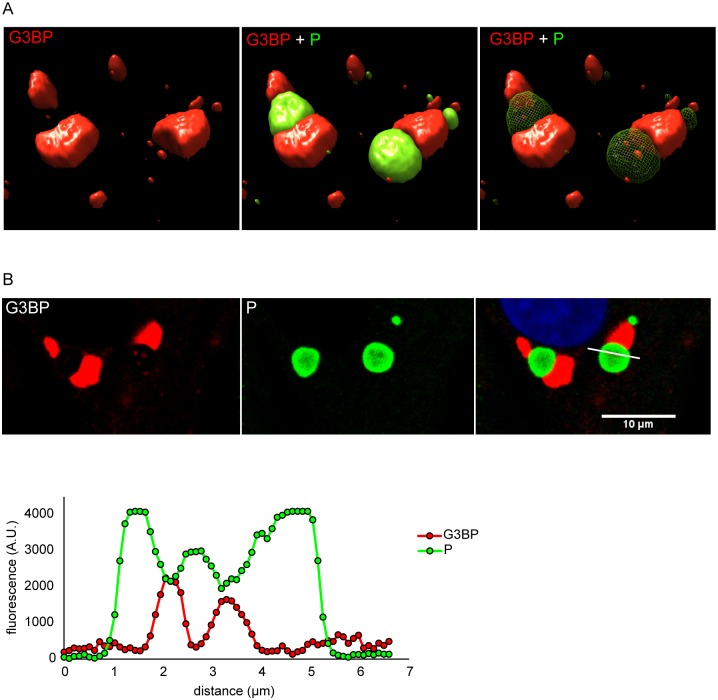
SGs are found adjacent to Negri bodies. Infected U373-MG cells were analyzed by confocal microscopy after staining with rabbit anti-P antibody (green) and mouse anti-G3BP1 antibody (red) followed by incubation with secondary fluorescent IgG as described above. (A) 3D analysis was performed as described in Material and Methods. (B) A focal plane extracted from the z-stack is displayed. The fluorescence corresponding to P (green) and G3BP (red) along the bar (upper panel) was quantified by using Image J software (lower panel).

In most of RABV-infected cells, SGs and NBs are in close proximity (Figs 1 and 2). To analyze more precisely the spatial relationship between both structures, we performed 3D analysis on cells infected for 24 h. Cells were immuno-stained for G3BP1 and RABV P protein to detect SGs and NBs, respectively. This analysis shows that SGs and NBs are two distinct but juxtaposed structures (Fig 6A). However, it is worth noting that some G3BP1-containing foci were enclosed inside the NBs, without any connection visible between these structures and the SGs that were surrounding the NBs. Moreover, these G3BP rich structures corresponded to NB areas where the P protein was less present (Fig 6B), excluding co-localization of both proteins. These results showed that G3BP components were present both around the NBs and in specific areas embedded within the NBs. They also indicated that SGs and viral factories can be very close but still remain distinct structures.

### Selective transport of viral RNA from NBs to SGs

**Fig 7:**
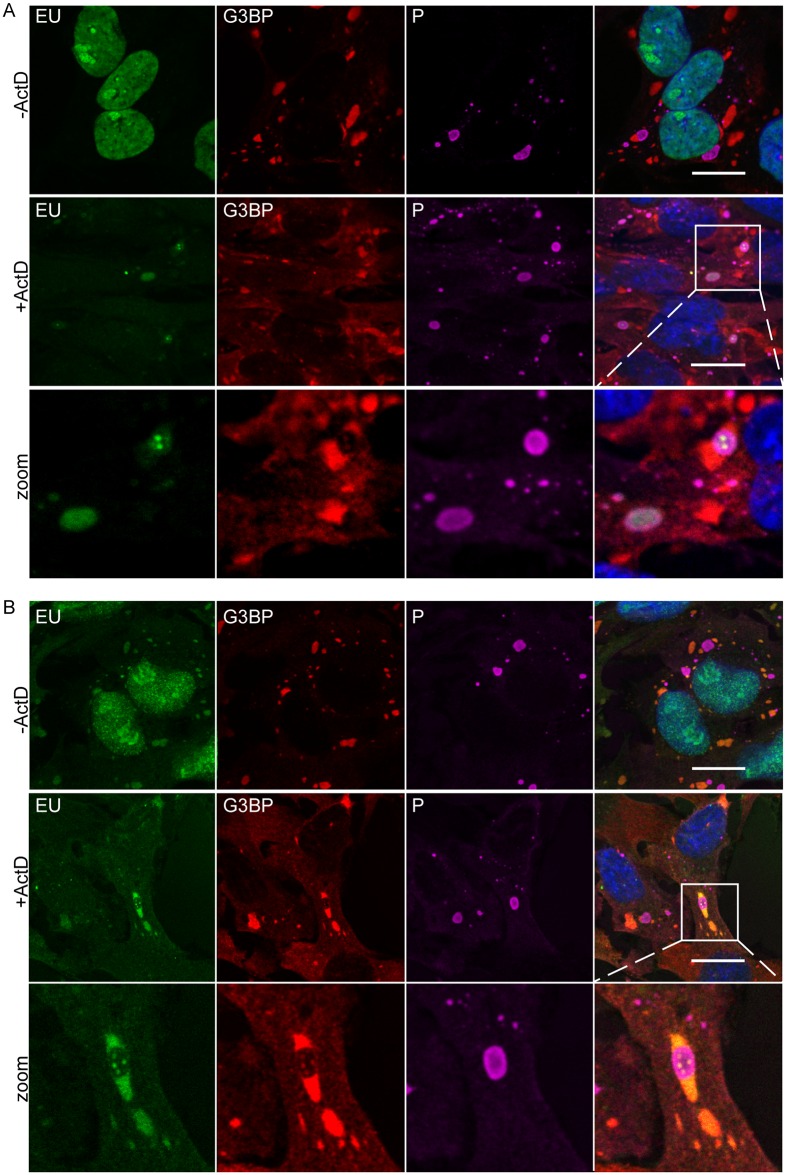
Localization of viral RNA and cellular RNA in SGs. (A) U373-MG were infected for 16 h then untreated (- Act D) or treated with 20 μM actinomycin D (+ Act D) for 1 h, to inhibit cellular transcription, and then fed with 1mM 5-ethynyl uridine (EU; Invitrogen) in a medium supplemented with (+Act D) or without Act D (- Act D) for 45 min. Cells were co-stained with the rabbit anti-P (purple) and mouse anti-G3BP (red) antibodies followed by incubation with secondary fluorescent IgG. Newly synthesized viral RNA was visualized by EU labeling of cells (green) as described in Materials and Methods. DAPI (blue) was used to stain the nuclei (merge). Magnification of the square in white is shown in the lower panel. The scale bars correspond to 15 μm. Colocalization of EU and G3BP was shown in the merge panel. (B) U373-MG were infected and treated as in (A). Then after EU labeling with (+Act D) or without Act D (- Act D) for 45 min, the medium containing EU was replaced by new culture medium supplemented with ActD (+ Act D) or without Act D (-Act D) for 3 h chase period. Cells were then analyzed as in Fig 7A. Magnification of the square in white is shown in the lower panel. The scale bars correspond to 15 μm. Colocalization of EU and G3BP was shown in the merge panel.

SGs are known to be RNA-rich granules [2]. As SGs and NBs are intimately associated, we explored whether viral RNA produced in NBs could localize to SGs in infected cells. We used 5-ethynyl uridine (EU) to perform short-term RNA labeling in presence of Actinomycin D (Act D). As Act D inhibits cellular transcription, only viral RNA was labeled. U373-MG cells were infected at a MOI of 3 for 20 h, before treatment or not with Act D for 1 h. Cells were then incubated in the presence of EU for 45 min. After fixation and permeabilization, cells were treated for detection of EU incorporation into nascent RNA and were simultaneously immuno-stained with anti-G3BP1 and RABV P antibodies for detection of SGs and NBs, respectively, for analysis by confocal microscopy. Without Act D, cellular RNAs were mainly detected in the nucleus, as expected (Fig 7A, upper panel). In presence of the drug, RABV RNAs were predominantly localized to NBs which are sites of viral transcription and replication (Fig 7A, middle panel) as previously shown [23]. In some viral factories, cell magnification revealed the presence of viral RNA as ponctate dots that sometimes corresponded to G3BP1-rich structures (Fig 7A, lower panel).

To analyze the fate of viral RNAs, we performed a pulse-chase analysis. Newly synthetized RNAs were labelled as described above. The cells were then incubated for 3 h in presence of Act D in new culture medium deprived of EU. In absence of Act D, EU labeling was essentially nuclear but RNA accumulation in the SGs was also detected (Fig 7B, upper panel). In cells treated with Act D, viral RNAs were mostly detected in SGs located in close proximity to NBs although some RNA dots were still present inside viral factories apparently associated with G3BP1-rich structures (Fig 7B, middle and lower panel). Taken together, these results indicated that viral RNAs were recruited from NBs to SGs.

**Fig 8:**
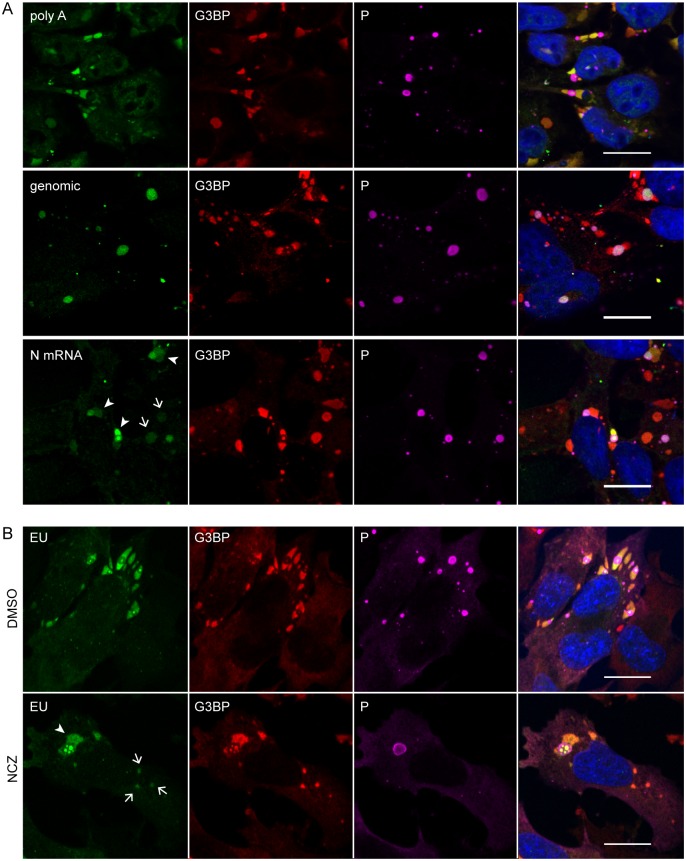
Viral mRNAs are specifically recruited to SGs and their transport from NBs to SGs does not require microtubules. (A) Cells were infected (MOI = 3) for 24 h. FISH experiments were performed by using 5’-ATTO448 modified oligonucleotides (Eurofins Genomics) to detect cellular mRNA (polyA), viral genomic RNA and N mRNA as described in Material and Methods. The proteins G3BP1 (red) and P (purple) were stained with the mouse anti-G3BP and rabbit anti-P antibodies followed by incubation with secondary fluorescent IgG. DAPI (blue) was used to stain the nuclei (merge). Note that viral mRNAs are detected in SGs close to NBs (arrowheads), but also in SGs far from NBs (arrows). The scale bars correspond to 15 μm. (B) U373-MG were infected for 16 h in presence of nocodazole (2 μM; NCZ) or mock-treated during viral infection. At 16 h p.i, cells were treated with 20 μM Act D and 1mM 5-ethynyl uridine (EU; Invitrogen) for 3 h. Cells were co-stained with the rabbit anti-P (purple) and mouse anti-G3BP (red) antibodies followed by incubation with secondary fluorescent IgG. Viral RNA was visualized by EU labeling of cells (green) as described in Materials and Methods. DAPI (blue) was used to stain the nuclei (merge). Note that viral mRNA are detected in SGs close NBs (arrowheads) but also in SGs far from NBs (arrows). The scale bars correspond to 15 μm.

To identify viral RNAs recruited in SGs, we performed fluorescent *in situ* hybridization (FISH) analysis by using specific oligonucleotide probes detecting either viral N mRNAs or viral genomic RNA and a poly-A probe detecting both cellular and viral mRNAs. Infected cells were simultaneously prepared for FISH and immuno-stained with anti-G3BP and anti-P antibodies. As expected, SGs contained *poly A+* mRNA that could have either cellular or viral origin. Viral mRNAs, such as the P mRNA, were detected in both NBs and SGs whereas viral genomic RNAs were localized exclusively in NBs (Fig 8A). These results indicated that viral mRNAs, but not genomic RNA, were selectively transported into SGs.

In order to determine whether the transport of viral RNA from NBs to SGs required microtubule network, infected cells were mock-treated or treated with nocodazole and viral RNA was detected by long-term EU labeling (for 3hours in presence of ActD). In both (mock or NCZ) conditions, viral RNA accumulated in SGs which most often are in contact with NBs (Fig 8B, arrowheads). Importantly, viral RNA was also detected in SGs which are far from NBs (Fig 8B, arrows). This result indicated that the transport of vRNA from NBs to SGs is independent of microtubule network.

### TIA-1 down-regulates RABV replication

**Fig 9:**
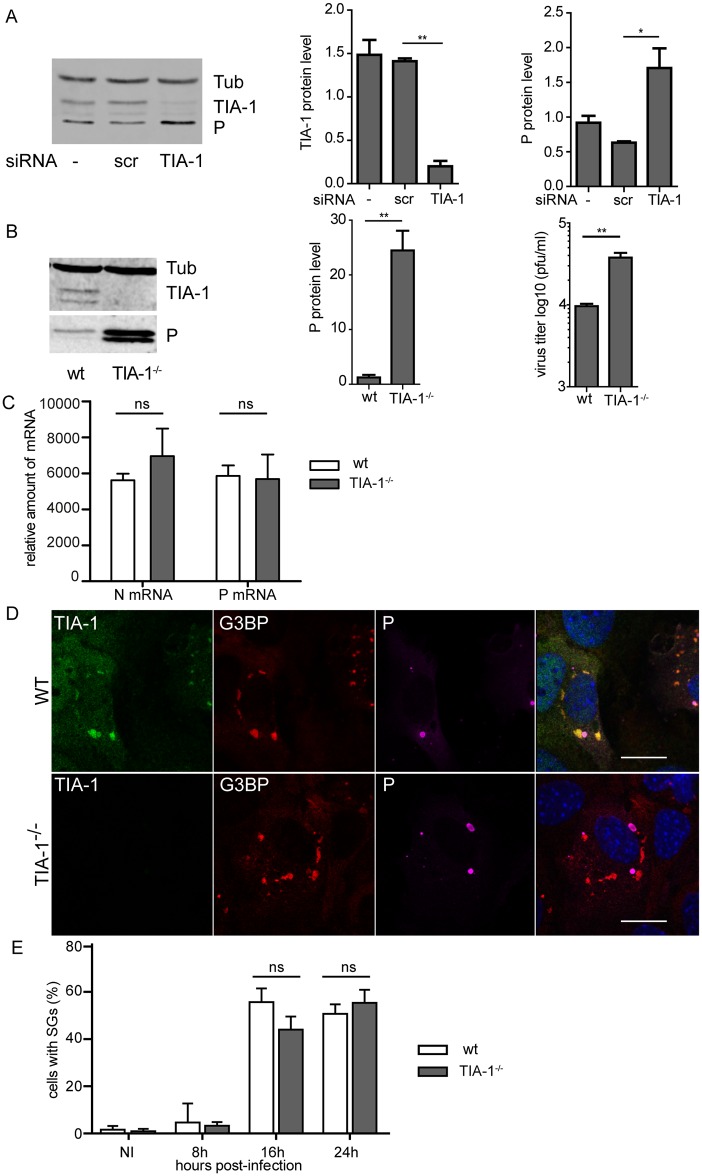
Effect of TIA-1 depletion on RABV infection. (A) U373-MG cells were non-transfected (-) or transfected with non-targeting (siScr) or TIA-1 targeting (siTIA) siRNA and 48 h p.i, cells were infected with RABV (MOI of 3) for 20 h. Cells extracts were analyzed by western-blot using anti-TIA-1, anti-P and anti-tubulin antibodies (left panel). Western blots from three independent experiments were quantified using immunoblot scanning and normalized with respect to the amount of Tub (middle and right panel). The amount of TIA-1 and P were measured in comparison to an arbitrary value of 1 applied to protein amounts obtained in untransfected cells. p values (* p <0.05) were determined using an unpaired Student *t* test. Error bars indicate the standard deviation. (B) WT MEFs and TIA-1^-/-^ MEFs were infected with RABV (MOI of 3) for 20 h. Cells extracts were analyzed by western-blot as in A (left panel) and quantification of P protein was performed as in A (middle panel). Supernatants from the cells were harvested for titration at 20 h p.i. Viral titers (PFU/ml) represent averages of titers from at least 3 independent experiments (right panel). (C) WT MEFs and TIA-1^-/-^ MEFs were infected as in (B), and total RNA were extracted at 20 h p.i. The relative amount of P mRNA and N mRNA were determined by RT-qPCR and normalized to those of GADPH mRNA as described in Material and Methods. Mean values and standard deviations of three independent experiments are shown. The difference in the level of P mRNA or N mRNA in WT MEF and TIA-1^-/-^ MEF are not statistically significant (ns). (D) WT MEFs (upper panel) and TIA-1^-/-^ MEFs (lower panel) were infected as in B. Cells were then stained with goat anti-TIA-1 (green), mouse anti-G3BP (red) and rabbit anti-P (purple) antibodies. DAPI (blue) was used to stain the nuclei (merge). Colocalization of TIA-1 and G3PB is apparent as yellow coloration in the merged panel. The scale bars correspond to 15 μm. (E) WT MEFs and TIA-1^-/-^ MEFs were infected with RABV for the indicated times. The percentage of cells containing SGs was quantified as described in Materials and Methods. Each time point represents the average of three independent experiments Error bars show standard deviations. The difference of the number of cells with SGs is not statistically significant (ns).

TIA-1, a major component of SGs has been described to be essential for their formation [31]. To investigate the role of TIA-1 in RABV replication, we analyzed the effect of down-regulation of TIA-1 by RNAi-mediated silencing. U373-MG cells were transfected with a pool of four siRNAs targeting TIA-1 (25 nM) or with non-targeting control (siScr) for 48 h, then cells were infected. Cells were harvested at 24 h post-infection and lysates analyzed by western blot to determine the expression of TIA-1, viral proteins and tubulin as control (Fig 9A). TIA-1 has two isoforms (TIA-1a 42 kDa and TIA-1b 40 kDa) generated by alternative splicing [32] and differentially expressed depending on the cell type [33]. In U373-MG cells, isoform expression ratio was in favor of TIA-1a (Fig 9A). As expected, TIA-1-targeting siRNAs inhibit TIA-1 protein expression in infected cells (around 80% of inhibition). TIA-1 depletion had no major effect on cell viability (>95%) measured by trypan blue exclusion, and resulted in a significant but moderate increase (around 2-fold) of the amount of the viral P protein at 24 h p.i. (Fig 9A), indicating that down regulation of TIA-1 expression resulted in an enhancement of viral protein expression.

These results led us to examine RABV infection in TIA1-knockout murine embryonic fibroblasts (TIA-1^-/-^MEFs) compared to wild-type MEFs (WT MEFs). Analysis of cell extracts by western blot confirmed the lack of TIA-1 expression in TIA1-knockout murine embryonic fibroblasts TIA-1^-/-^ MEFs, whereas WT MEFs expressed the two TIA-1 isoforms in roughly a 1:1 ratio (Fig 9B). In total absence of TIA-1, the viral P protein expression was very efficiently enhanced (around 20-fold), resulting in an increase of viral production (of ~5-fold) at 24 h p.i (Fig 9B). In contrast, similar mRNA transcript levels of RABV P and N genes were detected in TIA-1^-/-^ MEFs in comparison to control cells (Fig 9C). These data revealed that TIA-1 has an antiviral effect affecting the translation of viral proteins without modifying viral transcription.

We also analyzed whether the TIA-1 depletion impairs SGs formation. Strikingly, RABV-infected TIA^-/-^ MEFs exhibited formation of SGs as efficiently as WT MEFs did, as shown by G3BP1 staining (Fig 9D). Along this line, *poly A+* mRNA and viral mRNA were detected in SGs formed in both cell lines (S2 Fig). In addition, the kinetics of SGs formation during viral infection were similar in TIA^-/-^ MEFs compared to WT MEFs. In both cell lines, the number of infected cells containing SGs increased throughout the 24 h period of infection (Fig 9E). Furthermore, the SGs devoid of TIA-1 did not present any obvious morphological differences from the SGs formed in the WT MEFs. These results indicated that TIA-1 has little effect on the quantity of SGs and the kinetics of their formation.

We also examined whether TIA-1 was involved in the formation of SGs in a non-infectious context. The wild-type and TIA-1^-/-^ MEFs were treated with sodium arsenite to induce SGs formation. In both cell lines, G3BP1 formed similar aggregates (S3 Fig). This result is in contrast with a previous study demonstrating that the formation of SGs is significantly impaired in TIA-1^-/-^ MEFs [31], but supports recent findings showing that the impairment of SGs formation requires the concomitant depletion of TIA-1,TIAR and G3BP1 [34].

### Depletion of PKR prevents RABV-induced SGs formation, inhibits IFN-B gene expression and favors viral multiplication

**Fig 10:**
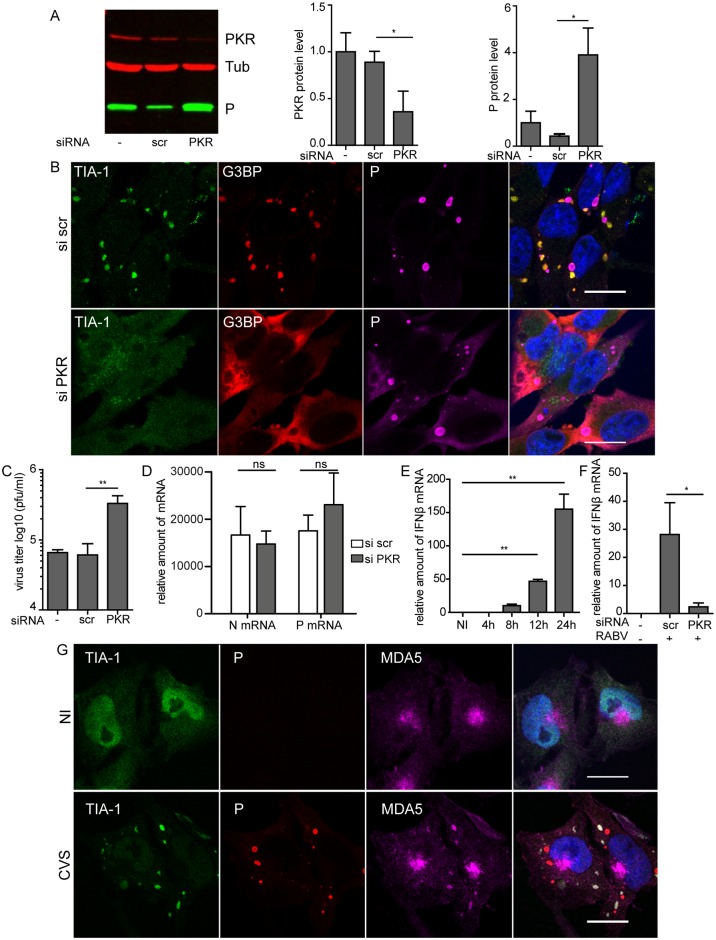
Effect of depletion of PKR on SGs and on RABV infection. (A) U373-MG cells were non-transfected (-) or transfected with non-targeting (siScr) or PKR-targeting (siPKR) siRNA and 48 h post-transfection, cells were infected with RABV (MOI of 3) for 20 h. Cell extracts were analyzed by western-blot using anti-PKR, anti-P and anti-α-tubulin antibodies (left panel). Western blots from three independent experiments were quantified using immunoblot scanning and normalized with respect to the amount of Tubulin (middle and right panel). p values were determined as described in Fig 8A. (B) U373-MG cells were transfected with non-targeting (siScr) or PKR targeting (siPKR) siRNA and 48 h post-transfection cells were infected with RABV (MOI of 3) for 20 h. Cells were then stained with goat anti-TIA-1 (green), mouse anti-G3BP (red) and rabbit anti-P (purple) antibodies as described in Fig 1. DAPI (blue) was used to stain the nuclei (merge). Colocalization of TIA-1 and G3BP is apparent as yellow coloration in the merged panel. The scale bars correspond to 15 μm. (C) Supernatants from cells in A were harvested for titration at 20 h p.i. Viral titers (PFU/ml) represent averages of titers from at least 3 independent experiments. (**p value <0.01). Error bars indicate the standard deviation. (D) Cells were treated as in (A) and total RNA were extracted at 20 h p.i. The relative amounts of P mRNA and N mRNA were determined by RT-qPCR and normalized to those of GADPH mRNA as described in Material and Methods. Mean values and standard deviations of three independent experiments are shown (ns for no statistical differences). (E) U373-MG cells were infected with RABV (MOI of 3) for the indicated times pi. Total RNA were extracted and the relative amounts of IFN-B mRNA were determined by RT-qPCR, and normalized to those of GADPH mRNA. Each time point represents the average of three independent experiments. p values (* p < 0.01) were determined using a paired Student *t* test. Error bars show standard deviations. (F) U373-MG cells were non-transfected (-) or transfected with non-targeting (siScr) or PKR targeting (siPKR) siRNA and 48 h post-transfection, cells were infected with RABV (MOI of 3) for 20 h. After RNA extraction, the relative amounts of IFN-B mRNA were determined by RT-qPCR, and normalized to those of GADPH mRNA as described in Material and Methods. Mean values and standard deviations of three independent experiments are shown. P values (* p <0.05) were determined using a paired Student *t* test. Error bars indicate the standard deviation. (G) Detection of MDA5 in RABV-induced SGs. U373-MG cells were non infected (NI) or infected with CVS (MOI of 3) for 20 h. Cells were then stained with goat anti-TIA-1 (green), mouse anti-P (red) and rabbit anti-MDA5 (purple) antibodies. DAPI (blue) was used to stain the nuclei (merge). Colocalization is apparent as yellow coloration in the merged panel. The scale bars correspond to 15 μm.

We next determined whether PKR is the kinase responsible for SGs formation during RABV infection. Down-regulation of PKR expression was performed by small interfering (siRNA). U373-MG cells, previously transfected with a pool of four siRNAs targeting PKR (25 nM) or with non-targeting control (siScr) for 48 h, were infected for 20h. Silencing efficiency and viral protein expression were assessed by western blot analysis. PKR-targeting siRNA, but not control siRNA, clearly inhibited PKR expression in infected cells (around 50% to 70% of inhibition) (Fig 10A), PKR-silenced cells were then examined for the presence of SGs using indirect immunofluorescence for both SGs markers, TIA-1 and G3BP1 (Fig 10B). In infected siScr-treated cells, we observed a punctuate G3BP1 and TIA-1 staining pattern which is characteristic of SGs assembly in infected cells (see Fig 1A). In contrast, in infected siPKR-treated cells, G3BP1 remained mostly diffuse in the cytoplasm, TIA-1 localized in the nucleus, and, as a consequence, SGs were not detectable (Fig 10B). In cells treated with sodium arsenite, which induces SGs formation via HRI kinase [31], SG assembly was not impeded by siPKR treatment (S4 Fig). Taken together, these results indicate, that PKR is specifically required for SGs formation following RABV infection.

As SGs may play a role in antiviral responses, we have further analyzed the role of PKR depletion on RABV infection. A significant increase (4 to 5-fold) both in viral P protein expression (Fig 10A) and viral production (Fig 10C) was observed in PKR depleted cells in the absence of SGs. The fact that more P was located outside NBs in siPKR-treated cells could be due to the increase of P protein concentration as P (in contrast to N) was not exclusively located in NBs. In contrast, PKR silencing did not affect the mRNA transcript levels of RABV N and P genes in comparison to cells transfected by non-targeting siRNA used as control (Fig 10D). This indicated that PKR silencing led to increased translation of viral proteins without modifying viral transcription. These data demonstrated that PKR has an antiviral effect on RABV infection.

PKR, besides its role in inhibition of cellular translation and SGs formation, may also be important for type I IFN gene induction (essentially IFN-B gene) and modulation of antiviral innate immune responses [35–37]. As PKR displayed an antiviral effect in RABV-infected cells, we tested whether this kinase participates in IFN-B gene regulation. IFN-B gene expression was determined by qRT-PCR at different times post-infection. IFN β mRNAs were detected at 6 h p.i and gradually increased up to 24 h p.i (Fig 10E). In infected cells depleted for PKR, inhibition of PKR expression dramatically decreased the RABV-induced IFN-B gene expression (Fig 10F). These results showed that silencing of PKR resulted both in full inhibition of SGs formation and inhibition of IFN-B gene induction. To further investigate a potential link between stress pathway and IFN pathway, we focused on the RIG-I-like receptors such as RIG I and MDA5. Therefore, we analyzed their localization in RABV-infected cells by confocal analysis. MDA5 co-localized with TIA-1 in SGs (Fig 10G) whereas we detected no clear colocalization of RIG-I in SGs (S5 Fig). Taken together, these results indicate that RABV infection initiates PKR-dependent cellular stress pathways leading to formation of SGs and initiation of antiviral innate immune responses.

## Discussion

In this report, we show that RABV promotes the formation of stress granules in the cytoplasm of infected cells. The specific SGs components, G3BP1 and TIA-1, are recruited to RABV-induced SGs which also contain the SG-resident poly (A)-binding protein (PABP) [8].

### RABV-induced SGs are highly dynamic and in close contact with NBs

The SGs are detectable as soon as 6 h post-infection and the number of infected cells containing SGs increases throughout the infection. Their formation is a general process observed for different RABV strains and in different cell types (either neuronal or non-neuronal cells).

SGs formed in RABV-infected cells are distinct from the canonical SGs induced under stress conditions. First, their morphology and size are heterogeneous with an irregular shape compared to the canonical SGs. Second, their formation and dynamic are independent of an intact microtubule network (Fig 3) in contrast to canonical SGs which require microtubule integrity [26]. Microtubule-independent SGs formation has also been reported in the case of VSV-induced SGs which do not require an intact cytoskeleton for their formation [21]. Third, their maintenance during viral infection does not require viral and/or cellular translation, since cycloheximide treatment does not result in their disappearance in contrast to *bona fide* SGs (Fig 5A and 5B). Similar results have been observed for the “antiviral SGs” induced by vaccinia virus infection [38].

Real time imaging of cells expressing GFP-G3BP1 and infected with recombinant fluorescent RABV reveals that SGs are highly dynamic structures. First, small granules are formed throughout the cytoplasm; then, they fuse to form larger structures. Our data show that RABV-induced SGs undergo different fates over time: they can persist in infected cells or exhibit assembly/disassembly cycles (Fig 4). Our results indicate that RABV-induced SGs behave as liquid droplets. They are spherical and they grow over time by fusion events upon contact. In addition, they are composed of RNA and RNA-binding proteins, which as G3BP1, are enriched in intrinsically disordered and aggregation-prone domains and have been suggested to promote cytosolic phase transition [39]. Thus, our data are consistent with recent studies indicating that cytosolic liquid-phase transition is a general mechanism underlying the formation of RNA granules such as P bodies and SGs [28–30].

SGs induced by RABV are located close to cytoplasmic viral factories (NBs) in infected cells (Fig 6), similarly to antiviral SGs formed in vaccinia virus-infected cells [10, 38]. This is not the case of VSV-induced SG-like structures which co-localize or even share the same structure with the viral factories [21]. For RABV, SGs and viral factories appear as distinct structures, even when they are in close contact with one another. Indeed, in some cells, the SG marker G3BP1 is located inside the NBs in specific areas from which viral P proteins are excluded. Pulse-chase experiments reveal that viral mRNAs, although synthesized within NBs [23], are later detected in SGs. As shown by FISH analysis, both viral and cellular mRNAs accumulate in the SGs (Figs 7 and 8A). In contrast, no viral genomic RNA is detected in RABV-induced SGs. These findings indicate that viral mRNAs are specifically transported from NBs to SGs. This transport does not require microtubule network as nocodazole treatment has no effect on the accumulation of viral mRNAs in SGs (Fig 8B). However, we cannot exclude that a direct contact between NBs and SG is sufficient for the transfer of viral RNA in SGs. Indeed, both structures which are very dynamic might have been in contact at some point of infection. Since SGs have been proposed to be sites of mRNA sorting during translation inhibition, it is tempting to speculate that RABV mRNAs may be sequestered from the translation competent pools of mRNAs into SGs. Along this line, different species of viral RNA (messenger or genomic RNA, as well as unusual viral RNA species formed during RNA synthesis) have been detected in such compartments for positive or negative sense-RNA viruses [40–43].

### Evidence that RABV-mediated SGs are antiviral

The formation of *bona fide* SGs (formed upon arsenite treatment of cells) has been reported as being dependent on TIA-1, TIAR and G3BP proteins [44, 45]. Our results indicate that TIA-1 is not essential for the formation of arsenite-dependent SGs. Similarly, although TIA1 is recruited into SGs during viral infection, RABV-induced SGs do not require TIA-1 for their formation, as the SGs are formed efficiently in the absence of TIA-1 after infection of TIA-1^-/-^ MEFs or si-TIA-1-treated U373-MG cells (Fig 9D). This is correlated with recent findings showing that the impairment of SGs formation requires the concomitant depletion of TIA-1, TIAR and G3BP [34]. Although TIA-1 depletion does not inhibit SGs formation, it results in a positive effect on viral gene expression and viral production (Fig 9A and 9B) in RABV infected cells demonstrating the antiviral effect of TIA-1. This restrictive effect of TIA-1 has been described for VSV [21] and for several other viral infections [41]. It has been shown that TIA-1 inhibits VSV growth at the level of viral gene expression and/or replication [21]. We show here that TIA restricts RABV infection at the level of viral translation, emphasizing the antiviral function of TIA-1.

RABV-induced SGs are dependent on PKR expression as inhibition of PKR expression significantly prevents SGs formation (Fig 10). In this condition, viral protein expression and viral production increase indicating that PKR depletion favors viral replication (Fig 10A and 10C). In contrast, viral mRNA levels are unchanged in the absence of PKR (Fig 10D). These results are in accordance with the well-known role of PKR as an antiviral protein and a crucial sensor of viral infection whose activation results in phosphorylation of eIF2α and inhibition of translation initiation [11, 46]. As inhibition of PKR expression also dramatically decreases the RABV-induced IFN-B gene expression (Fig 10E), our results indicate that PKR has a pivotal role in the initiation of cellular stress pathways leading to formation of stress granules and antiviral innate immune responses. Multifaceted roles of PKR in the regulation of stress granule formation and virus-induced gene regulation have been documented in recent years [25, 36, 47–49]. PKR has also been reported to be located in virus-induced SGs where it colocalizes with viral RNA sensors RIG-I and MDA5 in order to promote their interaction with different forms of viral RNA, suggesting an antiviral role for SGs [8, 49]. The inhibitory effect of PKR depletion on SGs formation, increased phosphorylation of eIF2α subunit in RABV-infected cells and concomitant decrease in IFN-B gene expression levels indicate that SG-dependent and PKR-mediated antiviral response is triggered during RABV infection. This is consistent with the presence of MDA5 in RABV-induced SGs (Fig 10G) suggesting that SGs may function as a scaffold for viral RNA recognition by RLRs. Our results altogether provide some evidence that RABV induces the formation of SG-like antiviral structures as shown for several viruses [8, 38].

### Is there any benefit for RABV to induce SGs?

Viral infections have been shown to induce [42, 50] or suppress SGs assembly [17, 51]. The induction is often only transient and followed, at some point of the infection, by SGs disassembly [18, 52, 53], apparition of new SGs modified in their composition [19, 20] or subversion of the SGs components for replicative advantages [10, 54]. Our data indicate that RABV induces the formation of SGs that persist at later times of infection. Although we did not get the conditions which totally inhibit SG formation (except by PKR depletion as discussed above), our data indicate that RABV infection is efficient in SG-positive cells despite the described antiviral role of SGs. The sequestration of a pool of viral mRNAs in the SGs suggests that the virus uses the SGs to control the amount of viral transcripts to modulate viral transcription and replication. This may limit the cytopathic effect and consequently the cellular damage. However, we cannot exclude that the SG-mediated antiviral responses are counteracted by the virus. We have previously shown that RABV P protein interacts with STAT proteins, inhibits their translocation to the nucleus and prevents STAT-mediated transcription of ISG [55–57]. As IFN-induced PKR expression contributes to the amplification of PKR-mediated antiviral immunity, RABV may block PKR- and SG-mediated antiviral responses through P-STAT interactions. Other data link the stress response pathway to the interferon response by demonstrating localization of PRR in SGs following infection [9, 25, 43, 58]. It has been very recently shown that the SGs induced by Newcastle disease virus, another member of the *Mononegavirales* order, recruit vRNA and RIG-I which trigger the induction of IFN cooperating in an efficient antiviral program [47]. Whether the ability of RABV to tolerate SG formation is due to anti-IFN effects of RABV proteins or to a beneficial role of SGs in viral infection requires further investigations.

## Materials and Methods

### Cells and viruses

BSR cells, cloned from BHK 21 (baby hamster kidney) were obtained from A. Flamand (I2BC, Département de Virologie, former Laboratoire de Génétique des Virus, Gif, France), N2A cells (mouse neuroblastoma) and U373-MG cells (human gliobastoma astrocytoma) were purchased from the ATTC organization (http://www.lgcstandards-atcc.org). All the cells were grown in Dulbeco’s modified eagle medium (DMEM) supplemented with 10% FCS (fetal calf serum). Immortalized murine embryonic fibroblasts (MEFs) from wild-type (WT) and TIA1-knockout mice [59] were obtained from P. Anderson (Harvard University). Mouse primary neurons were obtained from JM. Peyrin (Université Pierre-et-Marie Curie, CNRS UMR 7102, Paris) as described [60]. Briefly, cortical cells were isolated from mouse embryos E16 embryos of Swiss mice (Janvier Labs), and cultured for 5 days in complete neuronal culture medium DMEM glutamax (Life Technologies, Inc., Gaithersburg, MD, USA) supplemented with serum-free Neurobasal (Gibco) and 2% B-27 supplement (Gibco).

The SADB19, PV (Pasteur Virus), and CVS (Chalenge virus standard) strains of rabies virus were grown in BSR cells.

### Plasmids

The plasmid encoding G3BP-eGFP, described by [18] was kindly provided by R. Lloyd (Department of Molecular Virology and Microbiology, Baylor College of Medicine, Houston, USA).

### Construction and recovery of the recombinant CVS N2C virus expressing a P-mCherry fusion protein

The full-length recombinant N2C (prCVSN2C) infectious clone was described previously [61]. The authentic P coding sequence was replaced with the P-mCherry fusion encoding sequence. The original full-length genomic plasmid was digested with AvrII and NruI restriction enzymes. Three overlapping fragments were amplified by PCR. The first one going from the AvrII site in the N gene to the end of the P coding sequence, the second one corresponding to the mCherry coding sequence and the third one going from the end of the P coding sequence to the NruI site in the G gene. The PCR products and the digested plasmid were assembled using Gibson Assembly kit (New England Biolabs) to obtain the resulting plasmid, prN2C-P-mCherry.

Recombinant viruses were recovered as described previously [62, 63]. Briefly, N2A cells (10^6^ cells) were transfected using lipofectamine 2000 (Invitrogen) with 0.85 μg of full-length prCVSN2C-P-mCherry, in addition to 0.4 μg pTIT-N, 0.2 μg pTIT-P, 0.2 μg pTIT-L and 0.15 μg pTIT-G, which encode respectively the N, P, L and G proteins of SAD-L16 rabies virus strain. These plasmids were cotransfected with 0.25 μg of a plasmid encoding the T7 RNA polymerase. Six days posttransfection, the supernatant was passaged on fresh N2A cells, and infectious recombinant viruses were detected three days later by the fluorescence of the P-mCherry protein.

### Antibodies and drugs

The rabbit polyclonal anti-P antibody was previously described [23]. The mouse monoclonal anti-G3BP-1 (2 F3) antibody was obtained from Sigma. The rabbit polyclonal anti-P antibody was previously described [23]. The rabbit anti-phospho eIF2α (04342) was obtained from Millipore. The Rabbit anti-MDA5 (33H12L34) was from Invitrogen, the rabbit anti-RIG-I (AT111) was from Enzo life; the goat monoclonal anti-TIA-1 (C20) and mouse anti-PABP (10E10) antibodies were from Santa-Cruz Biotechnology. Secondary fluorescent antibodies were purchased from Molecular Probes (Alexa fluor 488-, 568- or 647- conjugated) and Cell Signaling (Fluor 800 or Fluor 680 conjugated). Nocodazole (M1404), actinomycin D (A9415), sodiumarsenite (S7400) and cycloheximide (C7698) were obtained from Sigma.

### Immunofluorescence staining and confocal microscopy

Cells were fixed for 15 min with 4% PFA (paraformaldehyde) and permeabilized for 5 min with 0.1% Triton X-100 in PBS. Cells were incubated with the indicated primary antibodies for 1 h at RT, washed and incubated for 1h with Alexa fluor conjugated secondary antibodies. Following washing, cells were mounted with Vectashield (Vector labs) containing DAPI. Images were captured using a Leica SP8 confocal microscope (63X oil-immersion objective).

For 3D reconstruction, confocal stacks were treated with Chimera Software and 3D rendering was carried out using the volume viewer tool in Chimera Software.

### Quantification of stress granule positive cells

To determine the number of stress granule-positive cells three wide-field 20X images were captured per experiment. Cells displaying ponctate immunofluorescent foci of G3BP-1 were considered as stress granule positive. Counterstaining with anti-P antibody was performed to discriminate between infected and non-infected cells.

### Fluorescence *in situ* hybridization (FISH)

FISH was performed as previously described [23], with some modifications and by using 5’-ATTO448 modified oligonucleotides (Eurofins Genomics). The sequences of probes used to detect viral RNAs were previously described [23]. Messenger RNAs (mRNAs) were detected using 5’-ATTO488-oligo (dT). Cells infected with RABV were fixed in 4% PFA for 15 min at room temperature, permeabilized 5 min with 0.1% TX-100 in PBS, and incubated with primary and secondary antibodies as described above. After dehydration in 70% RNase-free ethanol overnight, coverslips were rehydrated in 2X SSC (1X SSC: 0.15M NaCl, 0.015 M sodium citrate) buffer, before prehybridization with 10 ng probe per coverslips in hybridization buffer (50% formamide (Sigma), 10% dextran sulfate sodium salt (Sigma), 20 μg/ml salmon sperm DNA (Invitrogen), 2X SSC) for 1 h at 60°C. Hybridization was carried out in hybridization buffer plus 10 ng probe per coverslips for 5 min at 60°C and 4 h at 37°C in the dark. Cells were washed in 2X SSC pre-warmed at 42°C and then fixed in 3.7% formaldehyde in PBS and mounted as described above.

### EU labelling

Infected cells (16 h p.i.) were treated with 20 μM actinomycin D (Act D) for 1h, to inhibit cellular transcription, and then fed with 1 mM 5-ethynyl uridine (EU; Invitrogen) for 45 min. For pulse-chase analyses, the medium containing EU was replaced by new culture medium supplemented with Act D throughout the chase period. Cells were fixed using 4% PFA in PBS. EU labeling of cells was detected according to the manufacturer’s instructions (Invitrogen, Click-it RNA imaging kits). After this step, cells were washed with PBS and incubated with antibodies as described above.

### Quantitative real-time RT-PCR (RT-qPCR)

U373 cells (2–4 10^5^) transfected by siRNA and infected with RABV-CVS for 24 h were flash frozen in liquid nitrogen and stored at -80°C. Total RNA was extracted with Nucleospin RNA II kit (Macherey Nagel, France) and 1 μg of RNA was used for first strand cDNA synthesis by reverse transcription with AffinityScript QPCR cDNA synthesis kit (Agilent Technologies, USA) and oligo(dT) primers (200 ng). The quantitative real-time PCR was performed on the Mx3000P apparatus (Stratagene, USA) in a total volume of 20 μl containing the first strand cDNA template, 200 nM of each primer and 1x Mesa green QPCR master mix plus solution (Eurogentec, France). Standard curves for Interferon-B gene (IFN-B) and the housekeeping gene glyceraldehyde-3-phosphate dehydrogenase (GAPDH) were obtained by using serial dilutions of human genomic DNA. The forward and reverse primers for mouse GADPH were: 5’-TCAACTACATGGTCTACATGTT-3’and 5’GGTCTCGCTCCTGGAAGAT-3’, respectively. Forward and reverse primers for human IFN-B and GAPDH genes were 5’- GTC TCC TCC AAA TTG CTC TC (f), 5’- ACA GGA GCT TCT GAC ACT GA (r), 5’- ACA GCC TCA AGA TCA TCA GC (f) and 5’- TCT TCT GGG TGG CAG TGA T (r), respectively. IFN-B mRNA levels were normalized to GAPDH expression levels that remained unaffected during viral infection. The plasmid pCW278 containing the cDNA of RABV-CVS-N2C genome was used to establish standard curves for quantification of viral N and P mRNAs and to calculate the amplification efficiency for each pair of primers used for RABV-N gene 5’- GCA GCA ATG CAG TTC TTT GA (f), 5’- GTC AAT TCC ATG CCT CCT GT (r), and RABV-P gene 5’- CTT GAG ATG GCC GAA GAG AC (f), 5’- ACG ATT GGA ACA GGA GGT TG (r), respectively. Each amplification reaction was carried out in triplicate with the following conditions: an initial denaturation at 95°C for 10 min, 40 cycles of 95°C for 10 s, 60°C for 30 s and 72°C for 10 s. The uniqueness and sizes of PCR products were checked by agarose gel electrophoresis. U373 cells mock-infected in parallel were used as controls.

### Small interfering RNA (siRNA) transfection

A pool of 4 siRNAs targeting PKR (EIF2AK2) or TIA-1 were purchased from GE Healthcare (ON-TARGET plus human EIF2AK2 siRNA SMART pool, ON-TARGET plus human TIA-1 siRNA SMART pool and ON-TARGET plus non-targeting pool). Cells were seeded at 5x10^4^ per well in 24 well plates the day before. Transient transfections were performed using Dharmafect reagent according to the manufacturer’s instructions. A final siRNA concentration of 25 nM was used. A second transfection was performed 24 h after the first one with the same siRNA concentration. Cells were infected 24 h after the second transfection. Western-blot and immunofluorescence analysis were performed 24 h post-infection. The treatment with sodium arsenite (0;5mM) was performed on non-infected cells 48 h post transfection.

### Western blot analysis

Cells were washed and re-suspended in PBS, lysed in hot Laemmli sample buffer and boiled for 5min. Proteins were separated by electrophoresis on 12% SDS-PAGE and transferred onto a nitrocellulose membrane. The membrane was blocked with 10% skimmed milk in TBS for 2 h and incubated overnight at 4°C with the corresponding antibodies. The blots were then washed extensively in TBS-0.5% Tween 20 and incubated for 1 h with Fluor 800-conjugated IgG or Fluor 680-conjugated IgG secondary antibody (Cell Signaling) at room temperature. After washing, the membranes were scanned with the Odyssey infrared imaging system (LI-COR, Lincoln, NE) at a wavelength of 700 or 800 nm. Protein spot levels were determined by using Image Studio software (LI-COR).

### Live cell microscopy

For live-cell imaging, U373 cells were seeded onto 35-mm micro-dishes (Ibidi) 24 h before transfection. Cells were transfected using Lipofectamine 2000 (Invitrogen) with a plasmid encoding G3BP-GFP. One hour after transfection cells were infected with CVS-N2C-P-mCherry rabies virus in DMEM FluoroBrite medium (Invitrogen) supplemented with 5% FCS. Live-cell time-lapse experiments were recovered with a Zeiss AxioObserver epifluorescence microscope (63X oil-immersion objective). Cells are maintained at 37°C and 5% CO_2_ during imaging.

## Supporting Information

S1 Movie Dynamics of RABV-induced SGs. U373-MG cells transiently expressing G3BP-GFP were infected with rCVSN2C-PmCherry. One cell, exhibiting a persistent pattern of SGs formation, was analyzed by time-lapse microscopy at 14 h p.i. One frame was taken every 1 min. G3BP-GFP signals (white) and P-mCherry signals (red) are merged, the time post-infection is displayed and the scale bar corresponds to 15 μm.
(AVI)

S2 Movie Dynamics of RABV-induced SGs. U373-MG cells transiently expressing G3BP-GFP were infected with rCVSN2C-PmCherry. One cell, exhibiting a transient pattern of SGs formation, was analyzed by time-lapse microscopy at 14 h p.i. as indicated for the S1 Movie.
(AVI)

S1 Fig Distribution of G3BP-GFP in uninfected and infected cells. U373-MG cells transiently expressing G3BP-GFP were uninfected or infected with rCVSN2C-PmCherry. G3BP is diffuse in the cytoplasm of non-infected cells and located in RABV-induced SGs.
(TIF)

S2 Fig TIA-1 depletion has no effect on the localization of viral mRNAs during viral infection. (A) WT MEF (upper panel) and TIA-1^-/-^ MEF (lower panel) were infected with RABV (MOI of 3) for 20 h. Cells were stained for G3BP (red) and P (purple) as in Fig 8. FISH was performed by using 5’-ATTO448 modified oligonucleotides (Eurofins Genomics) to detect cellular mRNA (polyA) (Green). (B) WT MEF (upper panel) and TIA-1^-/-^ MEF (lower panel) were treated with Act D (20μM) for 1 h and then fed with 1 mM 5-ethynyl uridine for 45 min. Cells were stained for G3BP (red) and P (purple) and newly synthesized viral RNA (green) was detected as in Fig 6A.
(TIF)

S3 Fig TIA-1 depletion has no effect on arsenite-induced SG formation. WT MEF (upper panel) and TIA-1^-/-^ MEF (lower panel) were untreated (NT) or treated with sodium arsenite (0.5 mM) for 30 min. Cells were then stained for TIA-1 and G3BP1 as above. DAPI (blue) was used to stain the nuclei (merge). Colocalization is apparent as yellow coloration in the merged panel. The scale bars correspond to 15 μm.
(TIF)

S4 Fig PKR depletion does not affect arsenite-induced SG formation. U373-MG cells were transfected with non-targeting (siScr) or PKR-targeting (siPKR) siRNA and treated with sodium arsenite (0.5mM). Cells were then stained for TIA-1 and G3BP1. DAPI (blue) was used to stain the nuclei (merge). Colocalization is apparent as yellow coloration in the merged panel. The scale bars correspond to 15 μm.
(TIF)

S5 Fig Localization of RIG-I in RABV-infected cells. U373-MG cells were infected with CVS (MOI of 3) for 20 h. Cells were then stained with a goat anti-TIA-1 (green), mouse anti-P (red) and the rabbit anti-RIG-I (purple). DAPI (blue) was used to stain the nuclei (merge). The scale bars correspond to 15 μm.
(TIF)
