# Supplementary figures and images for: Rabies Virus Infection Induces the Formation of Stress Granules Closely Connected to the Viral Factories
Source: PLoS Pathog. 2016 Oct 17;12(10):e1005942. doi: 10.1371/journal.ppat.1005942 (PMC5066959; doi:10.1371/journal.ppat.1005942)

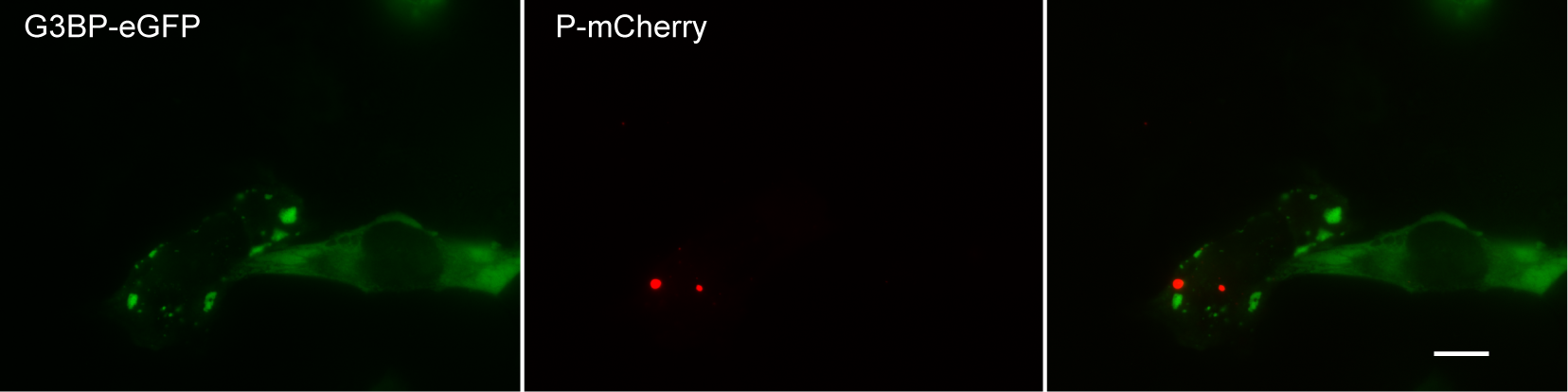

Supplement: S1 Fig — U373-MG cells transiently expressing G3BP-GFP were uninfected or infected with rCVSN2C-PmCherry. G3BP is diffuse in the cytoplasm of non-infected cells and located in RABV-induced SGs. (TIF) [file ppat.1005942.s003.tif]

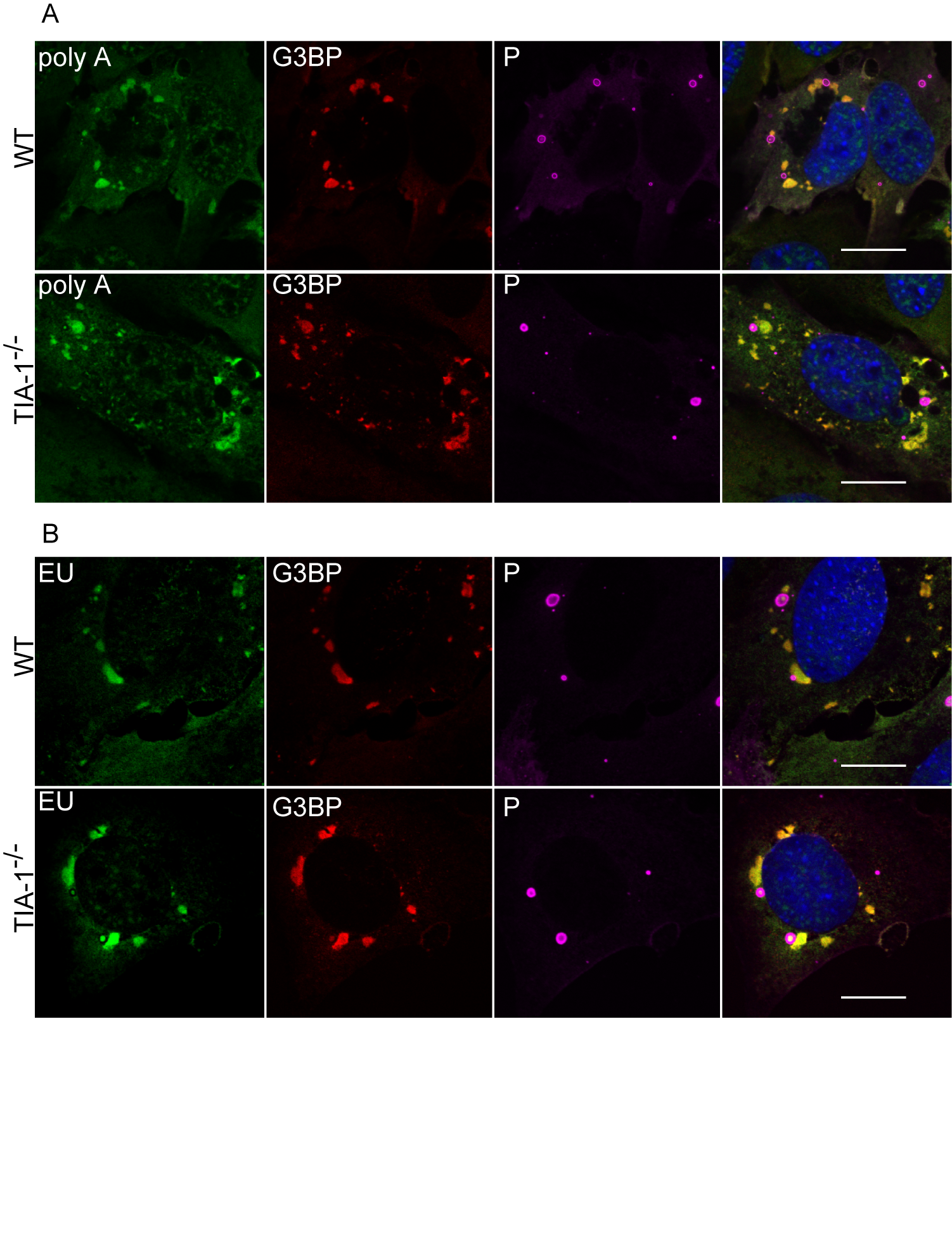

Supplement: S2 Fig — (A) WT MEF (upper panel) and TIA-1-/- MEF (lower panel) were infected with RABV (MOI of 3) for 20 h. Cells were stained for G3BP (red) and P (purple) as in Fig 8. FISH was performed by using 5’-ATTO448 modified oligonucleotides (Eurofins Genomics) to detect cellular mRNA (polyA) (Green). (B) WT MEF (upper panel) and TIA-1-/- MEF (lower panel) were treated with Act D (20μM) for 1 h and then fed with 1 mM 5-ethynyl uridine for 45 min. Cells were stained for G3BP (red) and P (purple) and newly synthesized viral RNA (green) was detected as in Fig 6A. (TIF) [file ppat.1005942.s004.tif]

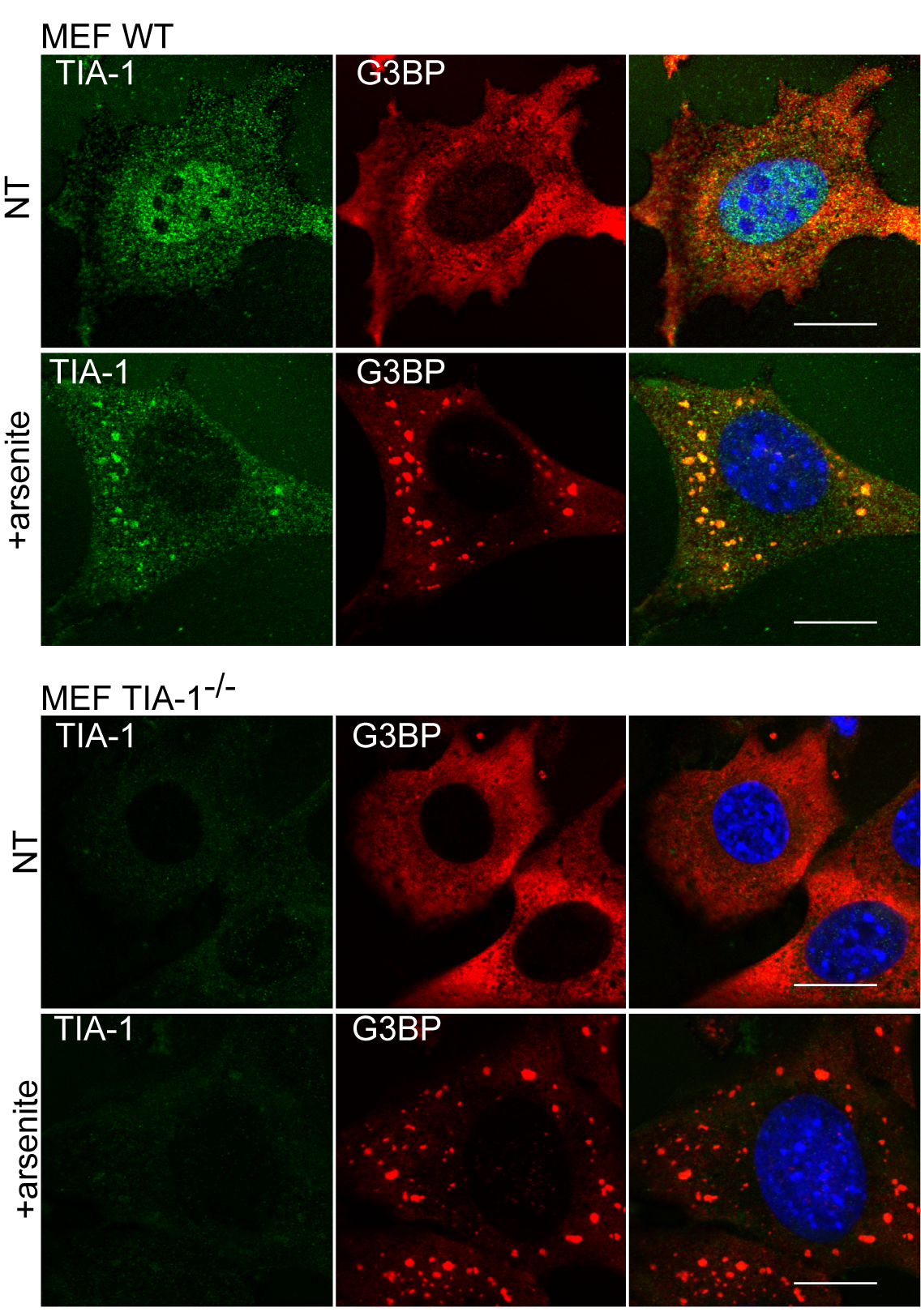

Supplement: S3 Fig — WT MEF (upper panel) and TIA-1-/- MEF (lower panel) were untreated (NT) or treated with sodium arsenite (0.5 mM) for 30 min. Cells were then stained for TIA-1 and G3BP1 as above. DAPI (blue) was used to stain the nuclei (merge). Colocalization is apparent as yellow coloration in the merged panel. The scale bars correspond to 15 μm. (TIF) [file ppat.1005942.s005.tif]

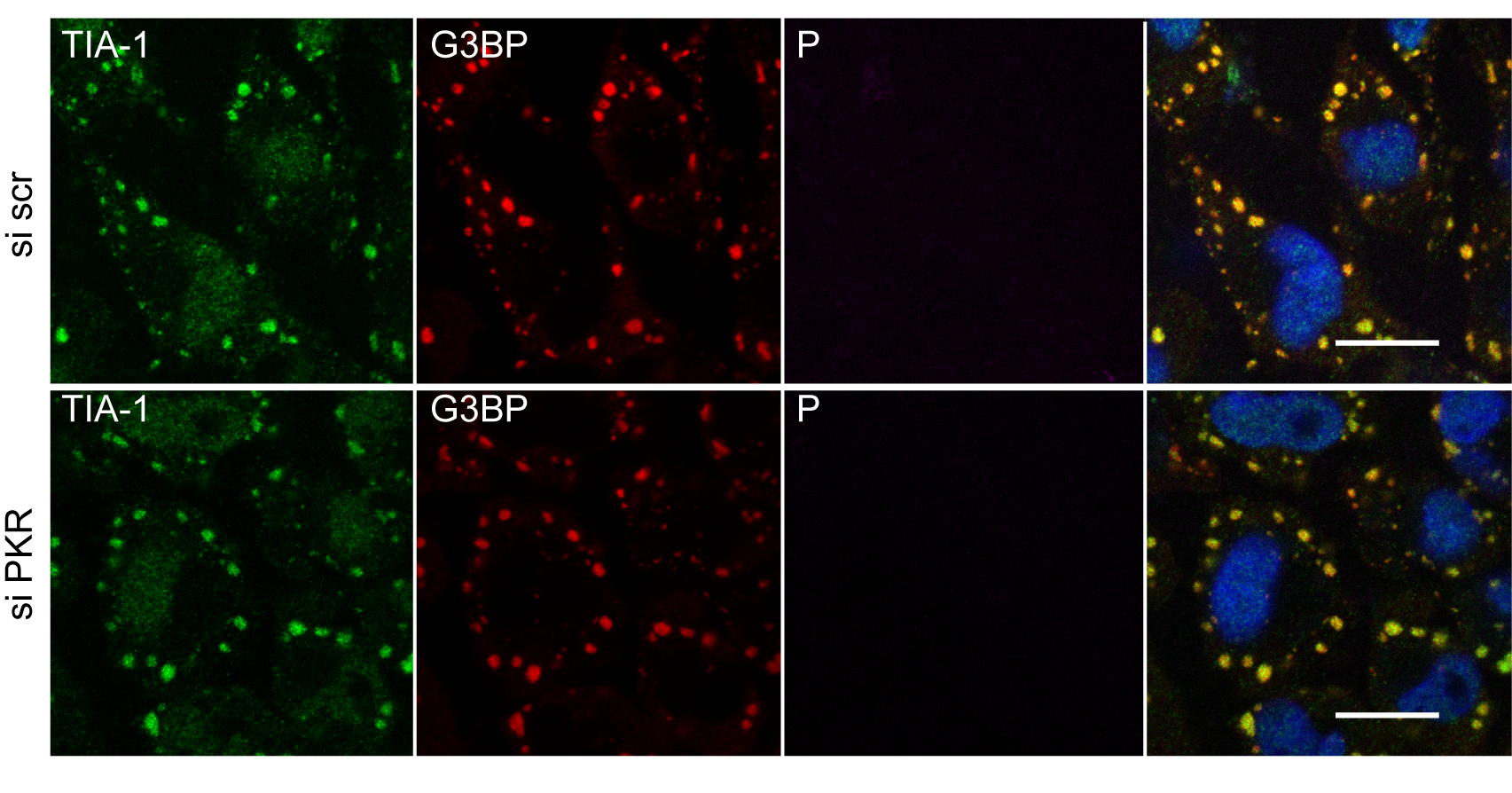

Supplement: S4 Fig — U373-MG cells were transfected with non-targeting (siScr) or PKR-targeting (siPKR) siRNA and treated with sodium arsenite (0.5mM). Cells were then stained for TIA-1 and G3BP1. DAPI (blue) was used to stain the nuclei (merge). Colocalization is apparent as yellow coloration in the merged panel. The scale bars correspond to 15 μm. (TIF) [file ppat.1005942.s006.tif]

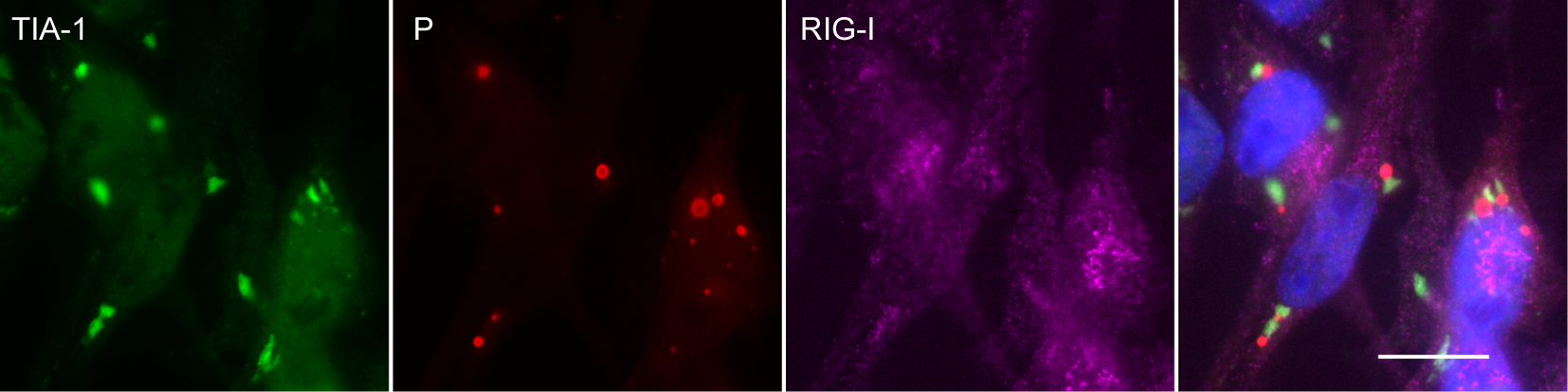

Supplement: S5 Fig — U373-MG cells were infected with CVS (MOI of 3) for 20 h. Cells were then stained with a goat anti-TIA-1 (green), mouse anti-P (red) and the rabbit anti-RIG-I (purple). DAPI (blue) was used to stain the nuclei (merge). The scale bars correspond to 15 μm. (TIF) [file ppat.1005942.s007.tif]
